# Mechanism of Changes in Goaf Water Hydrogeochemistry: A Case Study of the Menkeqing Coal Mine

**DOI:** 10.3390/ijerph20010536

**Published:** 2022-12-28

**Authors:** Xianming Zhao, Zhimin Xu, Yajun Sun

**Affiliations:** 1 School of Resources and Geosciences, China University of Mining and Technology, Xuzhou 221116, China; 2 Fundamental Research Laboratory for Mine Water Hazards Prevention and Controlling Technology, Xuzhou 221006, China

**Keywords:** goaf water, simulative experiment, hydrochemical characteristic, water–rock interaction, sulfate-reducing bacteria

## Abstract

Goaf water in mining areas is widely found in China’s coal mines. To clarify the hydrogeochemical characteristics of goaf water and the influence mechanism of water–rock interaction and further reveal microbial action on the formation of goaf water quality, the goaf water in the Menkeqing coal mine was taken as the object, and physical modeling was used to simulate the process of the real goaf changing from an oxygen-sufficient environment to an anoxic environment with the rise of groundwater level in this work. The experimental results showed that the water–rock interaction in the goaf was mainly the dissolution–precipitation of minerals in the rocks of the caving zone and fracture zone, cation exchange, and oxidation of pyrite in the coal layer. The primary sources of Na^+^ and K^+^ in the goaf water were the dissolution and reverse ion exchange of silicate minerals such as albite and potassium feldspar, while Ca^2+^ and Mg^2+^ mainly from the dissolution of minerals such as calcium feldspar, calcite, and chlorite. The oxidation of pyrite in coal was the main reason for the increase in SO_4_^2−^ concentration, the enhancement of reduction, and the decrease in pH and DO (dissolved oxygen) in the goaf water. Relative abundance of sulfate-reducing bacteria (SRB) in goaf (e.g., Desulfosporosinus, Desulfobacterium, etc.) increased gradually, inhibiting the increase in SO_4_^2−^ concentration in goaf water through the devulcanization of SRB. The inverse hydrogeochemical modeling was performed using PHREEQC for two stages of the simulation experiment: 0–30 days and 30–300 days. The simulation results show that the water–rock action in the formation of goaf water mainly occurred in the simulation experiment’s early stage (0–30 days), and the mineral dissolution is dominant throughout the experimental stage. The results of the study provide a theoretical reference for the prediction of highly mineralized water pollution in goaf and its prevention and control.

## 1. Introduction

In China, the exploitation method of coal resources is mainly underground mining [1,2]. Coal mining usually produces a large amount of mine water [3,4,5]. All equipment and materials are usually withdrawn, and the working panel is permanently closed within 45 days after mining is finished. Stopping drainage inevitably leads to a rise in the underground water level of the mine and forming goaf water [6,7]. Studies have shown that goaf water not only becomes a potential water hazard when the mine is in operation [8], but also becomes a contamination source of regional groundwater after the mine is closed [9,10]. Due to the influence of mining activities, the underground mining area becomes a gathering place for groundwater in the mining area, and also is a place where physical, chemical, and biological pollution of groundwater occurs [11,12]. After the groundwater is polluted by the goaf, it may continue to pollute the upper aquifers with the groundwater level rising. When the groundwater level exceeds the surface, polluted water will overflow and cause surface environmental pollution [13].

The quality of the goaf water is generally characterized by high sulfate content, high hardness, high total dissolved solids, and low pH [14]. Numerous studies have shown that the formation of goaf water hydrogeochemistry is mainly related to the mineral content of coal and caving rock [15,16,17]. Such as the oxidation of pyrite rich in coal or caving rock with water and air is the main reason for the increase in sulfate content and decrease in pH in the goaf water [14,18]. For mines in karst areas, high SO_4_^2−^ in goaf water is not just due to the oxidation of sulfides but also the acidic dissolution of gypsum [19]. Calcium in groundwater is exchanged for sodium in clay minerals, leading to the enrichment of sodium in goaf water [20]. Previous studies have shown that the existence of microorganisms accelerates the acidification process of mine water [21]. For example, thiobacillus is the main microorganism that reduces the pH of mine water by accelerating the oxidation leaching of pyrite [22]. These microorganisms mainly participate in the biological transformation process of sulfur elements and then affect acid water production. In summary, goaf water evolved from sulfides and other minerals by combining comprehensive physical, chemical, and biological processes [12].

In recent years, the coal mining center has been shifted from the Carboniferous coalfields in North China to the Jurassic coalfields in Northwest China. Many goaves have been produced and caused wide-range water seepage from the shallow layer into the subsurface coalmines, further polluting underground aquifers and exacerbating water shortages in arid and semi-arid regions [23,24]. Many scholars have developed a variety of water conservation mining technologies, such as filling mining [25], underground reservoirs [26], and deep well recharge [27], to address the problems of coal mining and groundwater resource protection in the fragile environment of arid regions. However, the above studies have mainly focused on the transport pathways of water resources affected by coal mining. Few systematic studies have been carried out on the mechanism of changes in goaf water hydrogeochemistry. This is due to the difficulty of long-term sampling and observation of mining water in the fissure zone after the closure of the mined area, resulting in few research reports on this subject.

Taking the Menkeqing Coal Mine in Northwest China as an example, this work investigates the formation conditions and mechanisms in the hydrochemical characteristic of goaf water in Jurassic coals from northwest China. This work has three objectives, (1) to simulate the generation process of the goaf water using the box simulation experiment; (2) to characterize the spatial and temporal variation of hydrochemical characteristics in goaf; (3) to identify the hydrogeochemical processes controlling the composition of goaf water. The research results are of great significance to predicting ion concentration changes in goaf water and accurately controlling groundwater pollution in the coal mine area.

## 2. The Geological Setting

**Figure 1:**
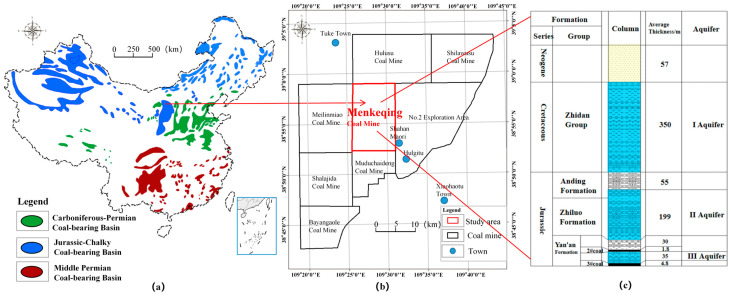
(**a**) Distribution of major coal resources in China; (**b**) location of Menkeqing Coal Mine; (**c**) stratigraphic histogram of Menkeqing Coal Mine.

As shown in Figure 1a,b, the Menkeqing Coal Mine is located in the Inner Mongolia Autonomous Region in the northwest of China and is a typical Jurassic coalfield. The coal layers in the field are nearly horizontal, and the main coal layer is the 3# coal seam. The stratigraphic histogram in the study area is shown in Figure 1c. The groundwater aquifers are divided from top to bottom as follows: Fourth Series loose rock pore diving aquifer, Cretaceous Lower Unified Zhidan Formation pore, and fissure bearing water aquifer (I aquifer); Jurassic Anding Formation, Zhiluo Formation to 2# coal seam roof fissure bearing water aquifer (II aquifer); 2# coal seam floor to 3# coal seam roof fissure bearing water aquifer (III aquifer). The Quaternary aquifer is a phreatic aquifer, and aquifers I, II, and III are confined aquifers. The goaf includes the caving zone and fracture zone [24,28]. According to the actual measurement, the caving zone and fracture zone produced by 3# coal seam mining are more than 70 m high, connecting the II and III aquifers. As the III aquifer is thin and its water abundance (specific capacity for 0.0058~0.0298 L/s·m) is weaker than that of the II aquifer (specific capacity for 0.1608~0.2068 L/s·m), the primary source of goaf water in the Menkeqing Coal Mine is the II aquifer.

## 3. Materials and Methods

Research on the chemical characteristics of groundwater is usually based on laboratory batch experiments and real site sampling tests [12,29]. However, it is difficult to sample the groundwater of real goaf and the simulation accuracy of batch experiments on real goaf is poor. Therefore, physical modeling is adopted in this work to simulate the variation characteristics of goaf water hydrogeochemistry in the process of changing from an oxygen-sufficient environment to an anoxic environment with the water level rising in goaf.

**Figure 2:**
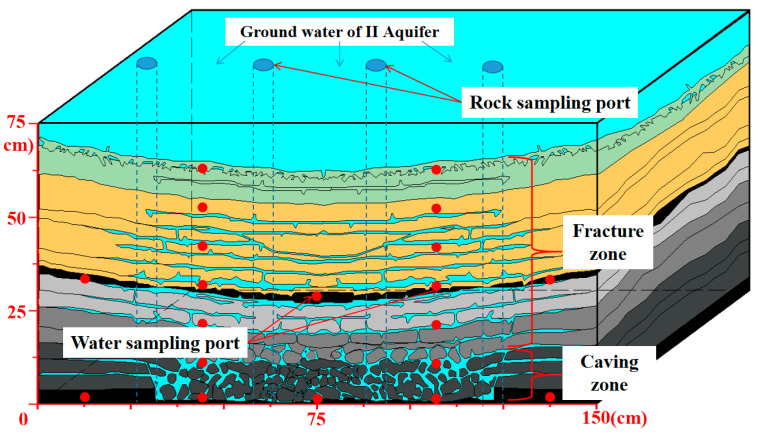
Schematic diagram showing the inflow, sampling ports, and goaf in the simulative box.

A 3D experimental setup (length, width, and height dimensions 150 × 75 × 75 cm, Figure 2) was made of transparent Plexiglas, with the lower layer (16.5 cm) simulating the caving zone of the goaf and the upper layer simulating the fissure zone of the goaf (53.5 cm). The caving zone consists of caving rock and 3# coal, and the fissure zone consists of fine sandstone, medium sandstone, sandy mudstone, mudstone, and 2# coal. All experiments’ rocks were taken from the rock layers of the Yan’an Formation of the Menkeqing Coal Mine. The relative thicknesses of the layers in the caving zone and fracture zone were determined from the stratigraphic column in Figure 1, and crack porosities were determined from a similar material test [30,31], as shown in Table 1 (similar material of test 3# coal seam working panel in the Menkeqing Coal Mine and binarization of fissures in goaf are shown in Figures S1 and S2 of the Supplementary Materials, respectively). The groundwater from the II aquifer was injected into the experimental device from the top to simulate the process of groundwater entering the goaf. The simulative box experiment lasted for 300 days. Figure 2 is a schematic drawing of the frontal view of the simulated goaf in physical modeling.

For monitoring the goaf water, 20 ports, 1 cm in diameter, were cut out of the Plexiglas wall to provide installation of the horizontal well as a water sampling port. Four simulative vertical wells were settled in the box as rock sampling ports, which penetrated the goaf’s thickness with 75 cm length and 4 cm diameter to allow coring of the caving zone and fracture zone.

### Subsec

According to the cross-sectional area (150 cm × 75 cm) of goaf and the average flow rate of groundwater in the II aquifer (0.3 cm/d), the influent water per day could be calculated (3375 mL/d). Then, a peristaltic pump was used to control the influent quantity, 2.34 mL/min. At the 10th, 20th, 30th, 60th, 90th, 120th, 150th, 180th, 210th, 240th and 300th days, the groundwater was sampled from the horizontal wells to monitor rgw hydrochemical characteristics of goaf water. Rock columns were removed from the vertical wells on the 30th, 90th, 180th, and 300th days. Then, each rock column was divided into seven parts corresponding to the goaf rock layers (3# coal, caving rock, fine sandstone, sandy mudstone, 2# coal, medium sandstone, mudstone) so that the mineral content changes i each rock layer during the simulative experiment could be analyzed. In addition, the composition of the microbial community attached to the rock surface in goaf could also be detected.

The groundwater from the II aquifer was loaded into five sterilized polyethylene drums (25 L), transported to the laboratory at −4 °C, and stored at −4 °C until injected into the experimental setup. The core of each rock layer was taken by drilling and processed into cuboids with a bottom surface of 4.5 cm × 4.5 cm and a length of 5 cm, 10 cm, 15 m, 20 m, and 35 m, respectively, to control the fissure rate of each rock layer. The fissure rate of the 2# coal seam, 3# coal seam, and caving rock was controlled by the mass of coal powder and rock block (density known). All rocks and coals were dried and sterilized by ultraviolet light for 30 min before being loaded into the experimental setup. Each water sample from the experimental setup was stored in two 50 mL polyethylene bottles and filtered through a 0.45 μm presterilized PES membrane. They were used to analyze the cations (the guaranteed reagent HNO_3_ was added, and the pH was adjusted below 2) and anions, respectively. A block rock sample of 1–2 cm in length and 2 cm in width, and no more than 15 mm in thickness, was selected from the surface of each rock layer in the experimental setup. The most smooth surface was selected as the test surface of mineral composition. Rock samples collected from the experimental setup were placed in sterile polyethylene bottles, then rinsed with sterile deionized water to shed biomass from the rock into the water, and finally immediately filtered through a 0.22 μm presterilized PES membrane to obtain biomass. The biomass was loaded into sterile Petri dishes and transported on dry ice to a molecular biology laboratory for DNA extraction.

Various instruments and methods measured the chemical and biological characteristics of the samples. The pH, ORP, and DO parameters of water samples were determined by the HI98195 multiparameter water quality analyzer (Hanna Instruments, Limena, Italy). HCO_3_^−^/CO_3_^2−^ was determined by titration (automatic potentiometric titrator, Metrohm, Herisau, Switzerland). An inductively coupled plasma emission spectrometer (ICP-OES, Spectro Analytical Instruments, Kleve, Germany) was used to detect Na^+^, K^+^, Ca^2+^, and Mg^2+^. SO_4_^2−^ and Cl^−^ were analyzed by ion chromatography (IC-925, Metrohm, Herisau, Switzerland). Total dissolved solids (TDS) were measured by the gravimetric method. In order to ensure the reliability of the data, the electroneutral error of all samples was less than 5%. The mineral content of the rock layers was analyzed by an X-ray diffractometer (D8 Advance, Bruker, Karlsruhe, Germany). DNA was extracted using an E.Z.N.A.^®^soil DNA kit (Omega Bio-Tek, Norcross, GA, USA). The quality of DNA extraction was checked on 1% agarose gel electrophoresis, and DNA concentration and purity were measured using a Nanodrop^®^ND-2000 UV–visible spectrophotometer (NanoDrop Technologies, Wilmington, DE, USA). Bacterial universal primers (338F and 806R) were used to amplify the V3-V4 segments of the extracted DNA (ABI GeneAmp^®^ 9700 PCR instrument). The amplification procedure was as follows: a 3 min predenaturation was performed at 95 °C, followed by 29 cycles of denaturation–annealing and extension procedures, and finally, a 10 min extension at 72 °C. The experiment was repeated three times for each sample, and the PCR products amplified from each sample were mixed. Then, the PCR amplification results were detected by 2% agarose gel electrophoresis. AxyPrepDNA gel recovery kits (AxygenBiosciences, Union City, CA, USA) were used to purify the PCR products. The purified and amplified fragments were sequenced by IlluminaMiseqPE300 (Shanghai Majorbio Technology Co., Ltd., Shanghai, China). The quality of the original sequence was filtered by Trimmomatic software, and the splicing and sequencing direction correction was performed by FLASH software. With 97% similarity, the sequences were OTU clustered using UPARSE software (version 7.0). Under a 70% comparison threshold, the species of each sequence were classified and commented on by the Ribosomal Database Project (RDP) classifier. At the same time, the classification of each 16S rRNA gene sequence was determined based on the 16S sequencing data by comparing with the Silva 16S rRNA database. By using the Tax4Fun function prediction tool, 16S taxonomic lineages in the Silva database were compared with the KEGG database to predict the function of microbial communities, and the KEGG Ontology (KO) number and abundance of genes were obtained. Series scatter charts, line charts, and half box-and-whisker diagrams were plotted using OriginPro 2023 software. The species’ relative abundance (genus level) of microbial samples was plotted using Ranguage 3.3.1. The PHREEQC Interactive 3.7.3 software was used to perform inverse hydrogeochemical modeling.

## 4. Results and Discussion

### 4.1. Changes in Mineral Content of Rock Layers in Goaf

With the rise of the goaf water level, goaf water contact with different rock strata in the caving zone and fracture zone for a long time. This will lead to dissolution, precipitation, ion exchange, and other water–rock interactions, resulting in the change of goaf water chemical characteristics [15,32]. X-ray diffractometer (XRD) was used to analyze the mineral content of various strata in the caving zone and fracture zone in the simulated goaf, which can reflect the types and contents of minerals in coal under the action of water–rock in the goaf. The analysis results are shown in Table 2. Quartz (SiO_2_) has the highest content in medium sandstone, followed by sandy mudstone and fine sandstone. Potassium feldspar (K_2_O·Al_2_O_3_·6SiO_2_) content is similar in fine sandstone, sandy mudstone, medium sandstone, and mudstone, but it is less distributed in caving rock and not detected in the coal. Plagioclase (Na(AlSi_3_O_8_)-Ca(Al_2_Si_2_O_8_)) is similar to potassium feldspar, with less weight content in coal and caving rock. In contrast, calcite (CaCO_3_) and pyrite (FeS_2_) are detected only in coal, and the pyrite content in 3# coal is significantly higher than in 2# coal. Siderite (FeCO_3_) is detected in small amounts in fine sandstone and medium sandstone. Among all clay minerals, kaolinite (Al_2_Si_2_O_5_(OH)_4_) has the highest content in coal, and illite (K_1.5_Al_4_(Si_6.5_Al_1.5_)O_20_(OH)_2_) and chlorite (Mg_2.5_Fe_2.5_Al_2_Si_3_O_10_(OH)_8_) are mainly distributed in other rock formations.

**Figure 3:**
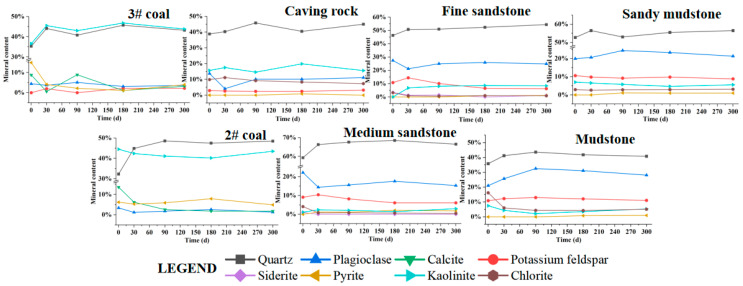
Mineral content changes in rock layers.

In the simulation experiment, the mineral content changes of each layer in the caving zone and fracture zone are shown in Figure 3. The relative content of stable minerals such as kaolinite and quartz in each layer is higher than at the beginning. The content of calcite and pyrite in 3# coal and 2# coal shows a relatively apparent decreasing trend, from the initial 9.8% and 10.2% relative content to 0.6% and 3.1%, respectively. The relative content of plagioclase in collapsed rock layers and medium sandstone layers shows a significant decreasing trend, while chlorite content in mudstone layers decreases most significantly, followed by medium sandstone layers. The variation of various minerals is the largest within 0–30 days at the beginning of the experiment. It then gradually becomes stable, which indicates that the weathering and precipitation of minerals mainly occur in the early stage of the formation of the goaf.

### 4.2. Changes in Hydrochemical Characteristics of Goaf Water

**Figure 4:**
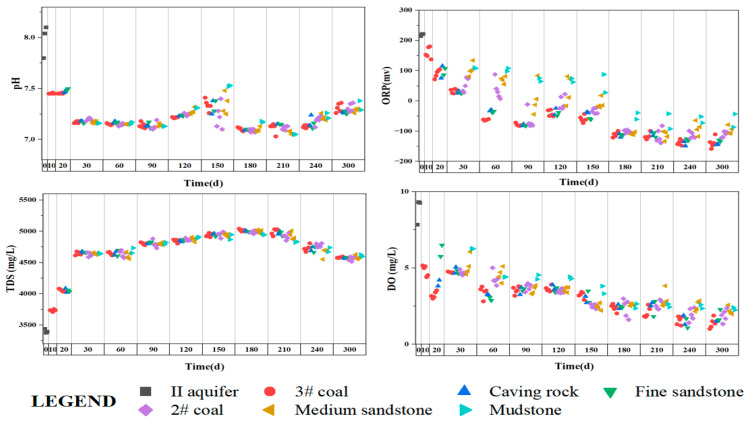
Spatiotemporal change scatter plots of pH, ORP, TDS, and DO.

The pH of groundwater is related to the geological environment in which it is located. For water accumulating in the goaf, the change in pH is a result of water–rock interaction and has an important influence on the changes of the components in the water [33]. O_2_ is a common oxidant in groundwater [34], and changes in DO concentration in water in a relatively closed extraction zone system can affect the type and intensity of water–rock interaction and the redox environment of the water body [35]. TDS represents the total amount of all solutes in groundwater and is a comprehensive reflection of the concentration of major ions in water [36]. Therefore, the pH, ORP, TDS, and DO of each rock formation water in the simulation experiment were tested, and the results are shown in Figure 4.

ORP, pH, and DO showed a significant negative correlation with TDS, indicating that the increase in TDS in the goaf water was related to mineral oxidation. At the beginning of the experiment (0–30 days), pH and DO decreased rapidly and then gradually stabilized. After 300 days of the experiment, the pH of the goaf water decreased from 8.01 to 7.26. DO decreased from 9.31 mg/L to 1.01 mg/L. The ORP also showed a gradual decrease and a negative value after 30 days, indicating that the goaf water changed from an oxidizing environment to a reducing environment during the experiment. In addition, DO of the water samples taken from the 3# coal layer was the lowest, and the reducibility was the strongest most of the time, while the mudstone layer at the top of the goaf was the opposite.

**Figure 5:**
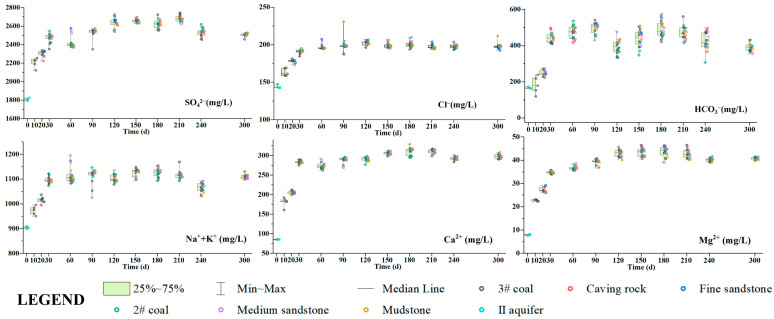
Half-box-and-whisker diagrams of changes in the concentration of ions.

**Figure 6:**
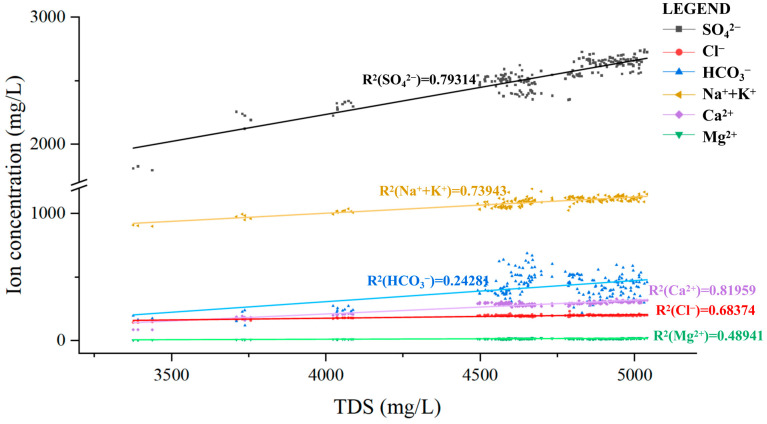
The relationship between TDS and ion concentration.

In this work, IC and ICP-OES were, respectively, used to determine the concentrations of major anions and cations in the experimentally collected water samples from each rock layer and have been plotted as half box-and-whisker diagrams, as shown in Figure 5. The magnitude of the cation mass concentration of the goaf water in the simulation experiment was Na^+^ + K^+^ > Ca^2+^ > Mg^2+^, and the anions were SO_4_^2−^ > HCO_3_^−^> Cl^−^. The concentrations of major anions and cations in the water samples of each stratum did not differ significantly, and they all increased rapidly at the beginning of the experiment (0–30 d) and gradually stabilized after reaching a peak after 30 days. The same is the mineral content change, indicating that water–rock interaction is mainly concentrated in the early stage of goaf water formation. However, there was no significant difference in ions in the water samples of different sampling layers at the same period (the specific spatiotemporal variation rule is shown in Table S1). As the fracture rate of the goaf increased compared with the original rock strata, the cross-sectional area of ion diffusion was expanded, thus intensifying the diffusion effect of ions in the goaf water. The linear correlation coefficient method was used to analyze the relationship between ion concentration and TDS of the goaf water, and the results are shown in Figure 6. In Figure 6, the concentration of each ion in the water samples increased with the increase in TDS, where R^2^(Na^+^ + K^+^) = 0.73943, R^2^(Ca^2+^) = 0.81959 and R^2^(SO_4_^2−^) = 0.79314, which indicates Na^+^ + K^+^, Ca^2+^, SO_4_^2−^, and TDS have a dominant positive correlation, so the experimental process of TDS is mainly related to the minerals containing these four elements. Combined with the changes in the mineral content of each rock layer in Section 4.1, it can be assumed that the dissolution of potassium feldspar, plagioclase, calcite, and other minerals, as well as pyrite oxidation, mainly occurred during the experiment.

### 4.3. Mechanisms Controlling Goaf Water Composition

#### 4.3.1. The Chemical Weathering Processes of Minerals

**Figure 7:**
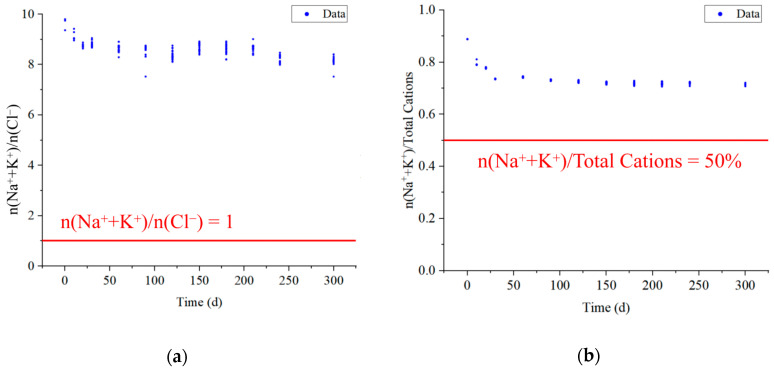
(**a**) Na^+^ + K^+^ versus Cl^−^ and (**b**) Na^+^ + K^+^ versus total cations scatter diagram.

In groundwater, Na^+^ and K^+^ may come from the dissolution of halite minerals (NaCl, KCl), hydrolysis of silicate minerals (albite, potassium feldspar, chlorite, etc.), and ion exchange [37,38]. The main source of Na^+^ and K^+^ in groundwater can be determined from the n(Na^+^ + K^+^)/n(Cl^−^) ratio [39]. As shown in Figure 7a, n(Na^+^ + K^+^)/n(Cl^−^) of water samples at all stages of the experiment were much greater than 1, indicating that Na^+^ + K^+^ was in excess relative to Cl^−^ and that dissolution of halite was not the main cause of the increase in Na^+^ + K^+^ concentration. Therefore, the primary sources of Na^+^ and K^+^ were silicate hydrolysis and a small part of ion exchange interaction.

Otherwise, the contribution of silicate dissolution to Na^+^ and K^+^ concentrations in groundwater can also be determined by the n(Na^+^ + K^+^)/total cation concentration (TCC) values. If n(Na^+^ + K^+^)/TCC > 50%, it indicates that Na+ and K+ groundwater is derived from silicate dissolution [40]. As shown in Figure 7b, n(Na^+^ + K^+^)/TCC is much greater than 50% for all water samples, which indicates that the hydrolysis of silicate minerals is the main reason for the increase in Na^+^ + K^+^ concentration in the goaf water. Combined with the analysis of mineral content and change characteristics in Section 4.1, the hydrolysis occurs mainly in plagioclase (albite) and potassium feldspar of collapsed rock layers and medium sandstone, and the reaction Equations (1) and (2) [41]. Otherwise, the albite mineral weathering also increases the HCO_3_^−^ concentration in goaf water.

(1) $$\begin{array}{c} 2{\mathrm{NaAlSi}}_{3}O_{8}+2{\mathrm{CO}}_{2}+3H_{2}O\\ \;\;\;\;\;\;\;\;\;\;\;\;\;\;\;\;\;\;\;\;\;\;\;\;\;\;\;\;\;\;\to {\mathrm{Al}}_{2}{\mathrm{Si}}_{2}O_{5}{\left(OH\right)}_{4}+2{\mathrm{Na}}^{+}+2{\mathrm{HCO}}_{3}^{-}+4{\mathrm{SiO}}_{2} \end{array}$$

(2) $$\begin{array}{c} 2{\mathrm{KAlSi}}_{3}O_{8}+2{\mathrm{CO}}_{2}+3H_{2}O\\ \;\;\;\;\;\;\;\;\;\;\;\;\;\;\;\;\;\;\;\;\;\;\;\;\;\;\;\;\;\;\to {\mathrm{Al}}_{2}{\mathrm{Si}}_{2}O_{5}{\left(OH\right)}_{4}+2K^{+}+2{\mathrm{HCO}}_{3}^{-}+4{\mathrm{SiO}}_{2} \end{array}$$

**Figure 8:**
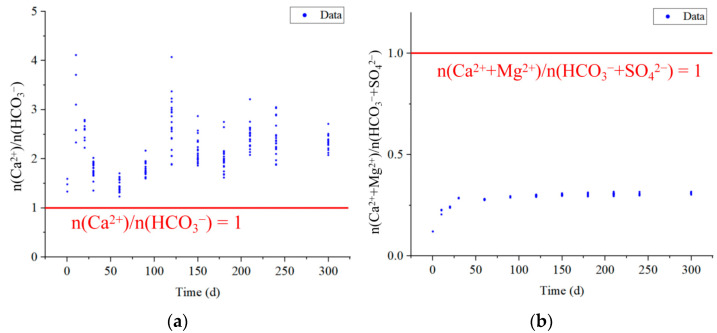
(**a**) Ca^2+^ versus HCO_3_^−^ and (**b**) Ca^2+^ + Mg^2+^ versus HCO_3_^−^ + SO_4_^2−^ scatter diagram.

The source of Ca^2+^ and Mg^2+^ in water can be determined comprehensively based on n(Ca^2+^)/n(HCO_3_^−^) ratio and (Ca^2+^ + Mg^2+^)/n(HCO_3_^−^ + SO_4_^2−^) ratio [39]. As shown in Figure 8a, n(Ca^2+^)/n(HCO_3_^−^) was greater than 1 for most of the experimental water samples, and Ca^2+^ was in excess relative to HCO_3_^−^, indicating that Ca^2+^ originated not only from the dissolution of calcite but also from the dissolution of silicate minerals [42].

In Figure 8b, the n(Ca^2+^ + Mg^2+^)/n(HCO_3_^−^ + SO_4_^2−^) values of the goaf water in the experiment are always less than 1, which also indicates carbonate weathering (3) and silicate weathering (4) were the dominant processes. Silicate weathering has occurred more strongly than carbonate weathering because all water samples have a ratio of less than 0.5. Combined with the characteristics of mineral content changes in Section 4.1, it can be judged that the main reason for the changes in Ca^2+^, Mg^2+^, and HCO_3_^−^ concentrations in the goaf water is the weathering of calcite in 2# coal and 3# coal layers, plagioclase (calcium feldspar) in caving rock and medium sandstone layers, and chlorite in mudstone and medium sandstone layers. The reaction Equations (3)–(5).

Furthermore, in the presence of HCO_3_^−^ in the reaction system, free Ca in the water undergoes a cation exchange reaction with Na on the mineral surface, displacing Na in the mineral, increasing the concentration of Na^+^ and reducing the concentration of Ca^2+^ in the effluent. From the perspective of the actual concentration change of Ca^2+^, the comprehensive effect of various effects is the increase in Ca^2+^ concentration, indicating that the sum of cation exchange, and adsorption is less than the dissolution of calcium-containing minerals in the experiment.

(3) $${\mathrm{CO}}_{2}+{\mathrm{CaCO}}_{3}+H_{2}O\to {\mathrm{Ca}}^{2+}+2{\mathrm{HCO}}_{3}^{-}$$

(4) $$\begin{array}{l} {\mathrm{CaAl}}_{2}{\mathrm{Si}}_{2}O_{8}+2{\mathrm{CO}}_{2}+3H_{2}O\\ \to {\mathrm{Al}}_{2}{\mathrm{Si}}_{2}O_{5}{\left(\mathrm{OH}\right)}_{4}+{\mathrm{Ca}}^{2+}+2{\mathrm{HCO}}_{3}^{-} \end{array}$$

(5) $$\begin{array}{l} \;{\mathrm{Mg}}_{2.5}{\mathrm{Fe}}_{2.5}{\mathrm{Al}}_{2}{\mathrm{Si}}_{3}O_{10}{\left(\mathrm{OH}\right)}_{8}+10{\mathrm{CO}}_{2}+5H_{2}O\to \\ \frac{5}{2}{\mathrm{Mg}}^{2+}+\frac{5}{2}{\mathrm{Fe}}^{2+}+{\mathrm{Al}}_{2}{\mathrm{Si}}_{2}O_{5}{\left(\mathrm{OH}\right)}_{4}+H_{4}{\mathrm{SiO}}_{4}+10{\mathrm{HCO}}_{3}^{-} \end{array}$$

Oxidative weathering of sulfide-bearing minerals, such as pyrite, and dissolution of (CaSO_4_) or gypsum (CaSO_4_·2H_2_O) release SO_4_^2−^ to groundwater. In the study area, pyrite only exists in the coal seams, and anhydrite and gypsum are not detected in the rock layers. Therefore, oxidation of pyrite leads to elevated SO_4_^2−^ in goaf water.

(6) $${\mathrm{FeS}}_{2}+\frac{7}{2}O_{2}+H_{2}O\to {\mathrm{Fe}}^{2+}+2{\mathrm{SO}}_{4}^{2-}+2H^{+}$$

**Figure 9:**
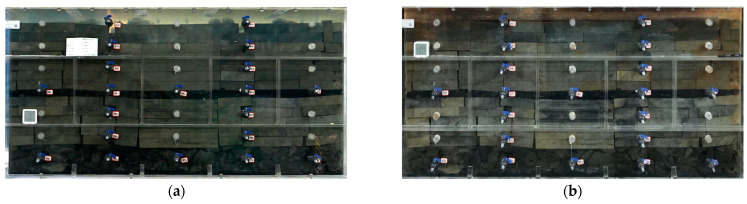
Color change of goaf water. (**a**) was taken on the 30th day of the experiment, and (**b**) was taken on the 300th day.

According to the changes of the conventional indexes of the goaf water in Section 4.2, it can be seen that the pH, DO, and ORP of the goaf water show a decreasing trend with time. At the bottom of the goaf water, the DO is the lowest and the strongest reduction simultaneously. There are two reasons for this phenomenon: firstly, as the goaf water level rises, the bottom of the goaf first becomes closed, making it difficult to contact the residual air at the top. Secondly, the 3# coal layer in the simulated goaf has the highest pyrite content. When the pyrite is in complete contact with water, pyrite undergoes oxidation to generate Fe^2+^ and SO_4_^2−^, which increases the concentration of SO_4_^2−^ in the goaf water, and then Fe^2+^ will be oxidized into Fe^3+^. Fe^3+^ will be used as the main oxidant to oxidize the pyrite to form More SO_4_^2−^, and the chemical reaction Equations (6)–(8) [43]. As shown in Figure 9, the color of goaf water in the model test device gradually changes to reddish-brown. The reason for this change is that Fe^2+^ produced by pyrite oxidation continues to oxidize to Fe^3+^, and Fe^3+^ combines with OH^−^ in alkaline goaf water to form Fe_(III)_ hydroxide (reddish-brown flocculated precipitate) [44]. Fe^2+^ can also be directly oxidized to ferric hydroxide when oxygen is sufficient, chemical reaction Equation (9), and causing the equilibrium of Equation (6) to shift to the right. This process continuously consumes oxygen in the water and produces reducing Fe^2+^ [45], resulting in lower DO and increased reduction at the bottom of the goaf water. In addition, the H^+^ produced during pyrite oxidation is the main reason for the overall pH decrease in the goaf water and further leads to silicate minerals’ dissolution.

(7) $${\mathrm{Fe}}^{2+}+H^{+}+\frac{1}{4}O_{2}\to {\mathrm{Fe}}^{3+}+\frac{1}{2}H_{2}O$$

(8) $${\mathrm{FeS}}_{2}+14{\mathrm{Fe}}^{3+}+8H_{2}O\to 15{\mathrm{Fe}}^{2+}+2{\mathrm{SO}}_{4}^{2-}+16H^{+}$$

(9) $$4{\mathrm{Fe}}^{2+}+O_{2}+2H_{2}O\to 4\mathrm{Fe}{\left(\mathrm{OH}\right)}_{3}↓$$

#### 4.3.2. Action of Biochemistry

**Figure 10:**
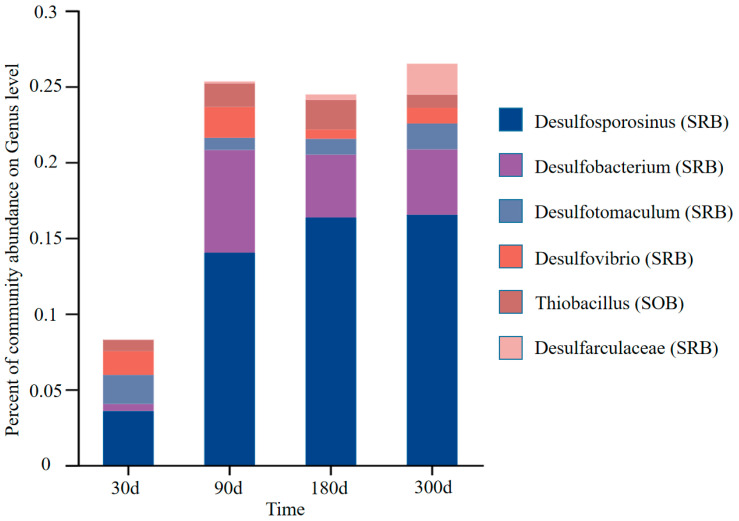
Variation characteristics of SRB and SOB community abundance during the experiment.

A large number of studies have shown that the changes in goaf water hydrogeochemistry may be related to the succession of microbial communities involved in sulfur metabolism, including sulfur-oxidizing bacteria (SOB) [46] and sulfate-reducing bacteria (SRB) [47]. As shown in Figure 10, during the simulation experiment, the relative abundance of SOB (Thiobacillus) attached to the rock surface in the goaf is 0.7~2%, much lower than that of SRBs (Desulfosporosinus, Desulfobacterium, Desulfotomaculum, Desulfovibrio, and Desulfarculaceae) 7.6~25.5%, indicating that SRB is the dominant bacteria in the simulated goaf. The trend of SRBs’ relative abundance was negatively correlated with DO and ORP. This is because most SRBs are anaerobic bacteria and are suitable to grow in a reducing environment.

**Figure 11:**
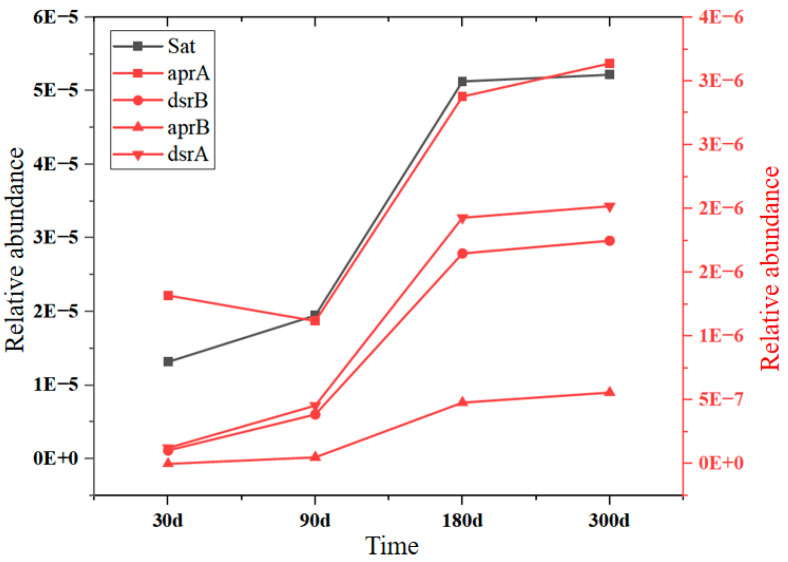
Variation characteristics sulfate reductive metabolic genes.

Devulcanization (sulfate respiration) is the primary way SRB affects hydrochemical characteristics [48]. In this way, SRB can reduce sulfate to sulfide such as H_2_S (Sulfide) and elevate the pH of water in goaf. Microbial sulfate reduction is mainly related to enzyme catalysis encoded by Sat, aprA, aprB, dsrA, and dsrB genes [49,50]. As shown in Figure 11, the relative abundance of all sulfate-reduction-related genes increased significantly from 90 to 180 days and slowed down from 180 to 300 days, indicating that the microbial sulfate reduction was significantly enhanced from 90 to 180 days and peaked after 180 days. Therefore, there are two reasons why the sulfate concentration in Figure 5 slows down or even decreases after 30 days of the experiment. On the one hand, DO decreases continuously in the closed environment of goaf, which leads to the gradual weakening of pyrite oxidation. On the other hand, in the anaerobic reduction environment, the relative abundance of sulfate-reducing bacteria increases gradually, which leads to the gradual strengthening of devulcanization, which is also the reason for the increase in pH in 90–300 days.

#### 4.3.3. Ion Exchange Process

Cation exchange can change the Ca^2+^ + Mg^2+^/Na^+^ + K^+^ ratio in groundwater, which causes the dissolution and precipitation of minerals [51]. The possibility of cation exchange can be inferred from the CAI-1 and CAI-2 indices in the Scholler method [52], which are calculated by Equations (10) and (11), respectively:

(10) $$\mathrm{CAI}-1=\frac{C\left({\mathrm{Cl}}^{-}\right)-\left[C\left({\mathrm{Na}}^{+}\right)+C\left(K^{+}\right)\right]}{C\left({\mathrm{Cl}}^{-}\right)}$$

(11) $$\mathrm{CAI}-2=\frac{C\left({\mathrm{Cl}}^{-}\right)-\left[C\left({\mathrm{Na}}^{+}\right)+C\left(K^{+}\right)\right]}{C\left({\mathrm{HCO}}_{3}^{-}\right)+C\left({\mathrm{SO}}_{4}^{-}\right)+C\left({\mathrm{CO}}_{3}^{-}\right)+C\left({\mathrm{NO}}_{3}^{-}\right)}$$

(12) $$2{\mathrm{Na}}^{+}+{\mathrm{CaX}}_{2}\to {\mathrm{Ca}}^{2+}+2\mathrm{NaX}$$

(13) $${\mathrm{Ca}}^{2+}+2\mathrm{NaX}\to 2{\mathrm{Na}}^{+}+{\mathrm{CaX}}_{2}$$

**Figure 12:**
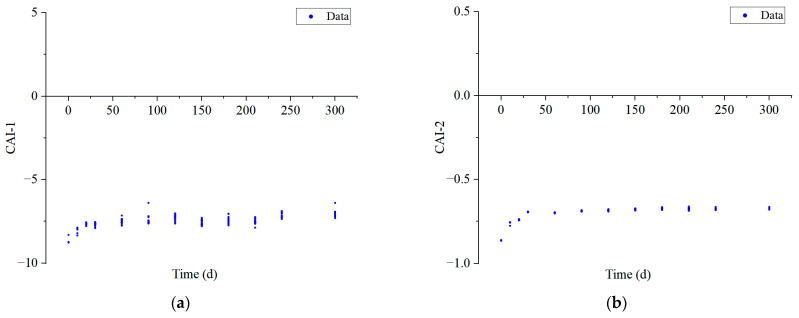
(**a**) CAI-1 and (**b**) CAI-2 indices of groundwater in goaf.

When the value of CAI-1 and CAI-2 is 0, it indicates that cation exchange does not occur. When CAI-1 and CAI-2 are all positive values, it suggests that there is a cation exchange between Ca^2+^ and Mg^2+^ in the coal mines material with Na^+^ and K^+^ in goaf water, reaction Equation (12). Otherwise, if values are negative, it suggests that reverse ion exchange occurs, and Ca^2+^ and Mg^2+^ in the goaf water are exchanged by the exchange of Na^+^ and K^+^ in the mineral reaction Equation (13), and the larger the absolute value of CAI-1 and CAI-2, the more intense the cation exchange. As shown in Figure 12, CAI-1 varied from −8.77 to −6.39, and CAI-2 varied from −0.86 to −0.66. Both the absolute values of CAI-1 and CAI-2 were small and negative, indicating that the reverse ion exchange reaction occurred in the system, and Ca^2+^ in the goaf water was exchanged by Na^+^ and K^+^ in the minerals, resulting in higher mass concentrations of Na^+^ and K^+^. As the experiment proceeded, the absolute values of CAI-1 and CAI-2 gradually decreased, indicating that the ion exchange reaction was strongest at the beginning of the experiment and gradually became weaker and reached equilibrium at the later stage.

#### 4.3.4. Inverse Geochemical Modeling

Reverse geochemical simulations can determine the water–rock reactions in the system based on the observed water chemistry information to determine the complex reaction conditions between groundwater and different minerals and gases and quantify them under reasonable conditions [53]. From Figure 4, the TDS changes more significantly during 0–30 days, while the tendency is smooth during 30–300 days. Therefore, we established a reverse geochemical model with these two stages as simulation paths to distinguish the difference between the water–rock interaction at the beginning and the end of the experiment. According to the mineral content analysis of each rock layer in the goaf in Section 4.1, we selected calcite, albite, potassium feldspar, pyrite, kaolinite, chlorite, quartz, and halite as the mineral phases involved in reactions. In addition, to more accurately describe the oxidation of pyrite and ion exchange, we added O_2_, CaX_2_, and NaX.

Based on the selected mineral phases, mass balance modeling was performed using PHREEQC to calculate the molar amounts of minerals transferred from the start to end points of the simulation paths. The calculated molar amounts of minerals transferred may vary due to differences among the selected potential mineral phases [54]. The modeling results with minimal residuals are summarized in Table 3.

According to Table 3, the absolute values of phase mole transfers in Simulation Path 1 are all greater than those of Simulation Path 2, which indicates that the hydrous rock action mainly occurred in the stage of 0–30 days. The weathering dissolution of calcite, albite, potassium feldspar, and chlorite mainly occurred in the system, while the precipitated minerals were kaolinite and quartz. The inverse simulation results are basically the same as those analyzed in Section 4.3.1 and Section 4.3.3. Otherwise, redox mole transfer also shows that pyrite oxidation mainly occurred in the early stage of the experiment, and this process consumed a large amount of oxygen in goaf water. The insufficient oxygen supply in the closed goaf became the main reason for inhibiting the continued oxidation of pyrite and the slight increase in pH.

In addition, the model results show that weak reverse ion exchange occurs in both stages, which is due to the lower Ca^2+^ equivalent concentration (average 14.76 meq/L) than Na^+^ (average 44.51 meq/L) in the goaf water, resulting in the difficulty of Ca^2+^ exchange in the goaf water by Na^+^ in the minerals. Therefore, compared with ion exchange, mineral weathering dominates the process of water quality change in the goaf.

## 5. Conclusions

Based on the above findings, the main conclusions are as follows:

During the experiment, the relative contents of stable minerals such as kaolinite and quartz in each rock layer of the caving zone and fracture zone in the simulated goaf increased relative to the initial, while the contents of plagioclase, calcite, pyrite, and chlorite in different rock layers showed an apparent decreasing trend. The changes of various minerals were the largest during 0–30 days at the beginning of the experiment and then gradually stabilized, which indicated that the weathering and precipitation of minerals in the goaf mainly occurred at the early stage of the formation of the goaf.

The pH and DO of the goaf water showed a decreasing trend, and the water body gradually changed from an oxidizing environment to a reducing environment. DO of the 3# coal layer at the bottom of the goaf is the smallest and is also the first area that changes from oxidizing to reducing. In addition, the main ion concentrations in the water in the mining area changed significantly, specifically Na^+^ + K^+^, Ca^2+^, HCO_3_^−^, and SO_4_^2−^ increased, and the concentrations of Mg^2+^ and Cl^−^ increased slightly. Na^+^ + K^+^ and SO_4_^2−^ contributed the most to the TDS of the goaf water and showed a significant linear positive correlation with TDS.

The main sources of Na^+^ and K^+^ in the goaf water were reverse ion exchange and the dissolution of silicate minerals such as albite and potassium feldspar, while Ca^2+^, Mg^2+^, and HCO_3_^−^ were mainly from the weathering of calcium feldspar, calcite, and chlorite. The oxidation of pyrite in the 3# coal layer at the bottom of the goaf was the main reason for the increase in SO_4_^2−^ concentration and reduction, decrease in pH and DO in the goaf water. The relative abundance of SRB in the experiment increased gradually and, through the devulcanization process, inhibited the increase in SO_4_^2−^ concentration in goaf water. Reverse geochemical simulation results showed that water–rock interaction occurred mainly in the early stage of the experiment (0–30 days) and that ion exchange was very weak compared to mineral weathering.

Altogether, this research provides new insight for elucidating the formation and evolution mechanism of goaf water hydrogeochemistry in the Menkeqing Coal Mine of Northwest China and developing groundwater pollution prediction and prevention technologies. More work should be conducted to explore the possibility of goaf water as an available groundwater resource in ecologically fragile mining areas and its potential harm in groundwater ecological restoration.

## Figures and Tables

**Table 1:** Parameters of rock layers in the physical modeling.

Goaf	Layers	Fissure Rate (%)	Thickness (cm)	Relative Level (cm)	Number of Sampling Ports
Caving zone	3# coal	22.25	0.5	0–0.5	5
	Caving rock		16	0.5–16.5	
Fracture zone	Fine sandstone	11.89	13.5	16.5–30	2
	Sandy mudstone	7.65	9	30–39	2
	2# coal	14.79	2	39–41	5
	Medium sandstone	7.12	20	41–61	4
	Mudstone	5.82	9	61–70	2

**Table 2:** Mineral content of rock layers.

Layers	Mineral Content (%)							Total Content of Clay Mineral (%)				
	Quartz	Potassium Feldspar	Plagioclase	Calcite	Siderite	Pyrite	Total Content of Clay Mineral	Illite/Smectite Mixed	Illite/Smectite Ratio	Illite	Kaolinite	Chlorite
3# coal	35.3	\	4.4	8.8	\	14.9	36.6	\	\	\	100	\
Caving rock	38.8	2.9	13.7	\	\	\	44.6	19	15	24	35	22
Fine sandstone	46.3	10.8	27.4	\	3.4		12.1	27	45	26	20	26
Sandy mudstone	52.7	10.6	19.9	\	\	\	16.8	20	15	21	42	17
2# coal	32.3	\	3.4	13.6	\	6.2	44.5	\	\	\	100	\
Medium sandstone	59.3	9.1	22.2	\	1.0	\	8.4	17	60	20	14	49
Mudstone	35.8	10.9	21.0	\	\	\	32.3	17	15	10	23	50

**Table 3:** Results of inverse geochemical modeling.

Simulation Path	Initial	Final	Phase Mole Transfers (mmol/L)											Redox Mole Transfers (mmol/L)	
			Calcite	Albite	Potassium Feldspar	Pyrite	Kaolinite	Chlorite	Quartz	Halite	O_2_(g)	CaX_2_	NaX	O(0)	S(2)
1	1 d	30 d	8.02	5.75	0.307	3.64	−0.21	0.23	−12.27	1.33	12.72	−0.34	0.68	25.45	7.27
2	30 d	300 d	0.37	1.51	0.086	0.79	−0.76	0.049	−2.91	0.19	2.76	−0.045	0.089	5.52	1.58

Positive values represent dissolution, while negative values represent precipitation.

## Data Availability

The data used to support the findings of this study are included within the article.

## Supplementary Materials

The following supporting information can be downloaded at: https://www.mdpi.com/article/10.3390/ijerph20010536/s1. Figure S1. (a) Similar material of test 3# coal seam working panel in Menkeqing Coal Mine(model scale 1:100), and (b) Goaf and fissures formed by mining 150 m. Figure S2. Binarization of fissures in similar material test. Table S1. Temporal and spatial variation in the goaf water hydrogeochemistry (Sample data mean value).
