# Supplementary material for: Mechanism of Changes in Goaf Water Hydrogeochemistry: A Case Study of the Menkeqing Coal Mine
Source: Int J Environ Res Public Health. 2022 Dec 28;20(1):536. doi: 10.3390/ijerph20010536 (PMC9819404; doi:10.3390/ijerph20010536)
Supplement: Supplementary file 1 [file ijerph-20-00536-s001.zip › ijerph-2093840-supplementary.pdf]

## Supplementary Materials:

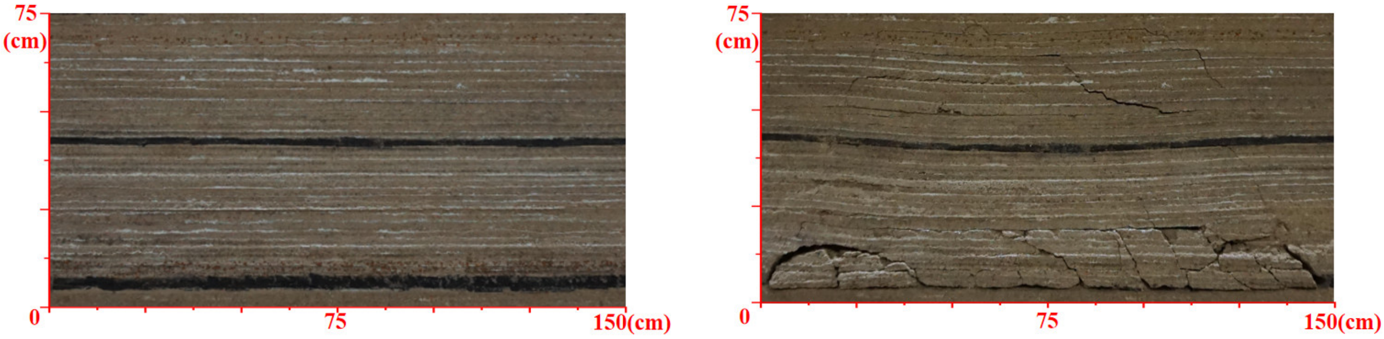


(a) (b)

**Figure S1.** （a）Similar material of test 3# coal seam working panel in Menkeqing Coal Mine(model scale 1:100), and （b） Goaf and fissures formed by mining 150m

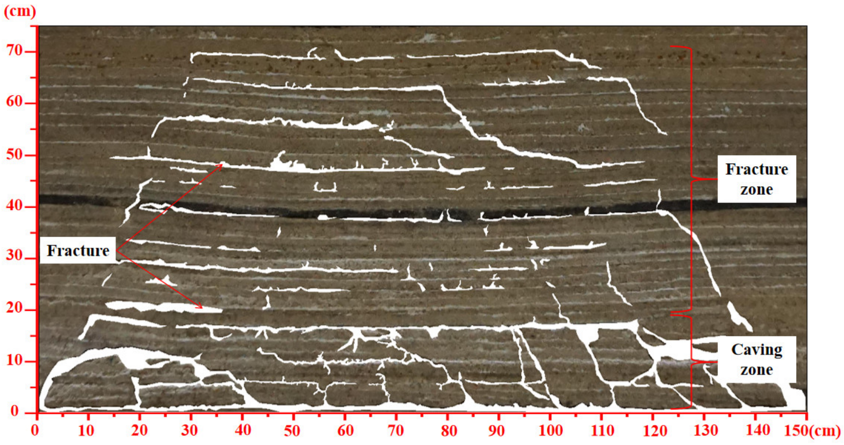


**Figure S2.** Binarization of fissures in similar material test

**Table S1.**Temporal and spatial variation in the goaf water hydrogeochemistry(Sample data mean value).

| Time (d) | Sample position (Layers) | pH | ORP (mv) | DO (mg/L) | TDS (mg/L) | $SO_4^{2-}$ (mg/L) | $Cl^-$ (mg/L) | $HCO_3^-$ (mg/L) | $Na^++K^+$ (mg/L) | $Ca^{2+}$ (mg/L) | $Mg^{2+}$ (mg/L) |
|---|---|---|---|---|---|---|---|---|---|---|---|
| 0 | | 7.98 | 218.50 | 8.80 | 3401.30 | 1808.78 | 144.42 | 167.21 | 904.19 | 85.50 | 7.89 |
| 10 | 3# coal | 7.45 | 159.32 | 4.84 | 3733.95 | 2206.19 | 163.41 | 181.55 | 972.95 | 180.93 | 22.73 |
| 20 | 3# coal | 7.44 | 91.16 | 3.24 | 4056.10 | 2318.19 | 179.41 | 246.47 | 1021.09 | 206.93 | 27.13 |
| | Caving rock | 7.47 | 95.50 | 4.01 | 4055.33 | 2260.16 | 175.40 | 256.56 | 1001.34 | 202.39 | 27.76 |
| | Fine sandstone | 7.49 | 96.80 | 6.12 | 4049.31 | 2301.56 | 177.39 | 249.27 | 1015.91 | 205.64 | 27.62 |
| 30 | 3# coal | 7.17 | 32.76 | 4.72 | 4644.17 | 2427.15 | 188.47 | 415.30 | 1092.99 | 283.19 | 34.57 |
| | Caving rock | 7.18 | 29.30 | 4.86 | 4666.79 | 2470.65 | 191.05 | 494.05 | 1095.92 | 284.35 | 34.92 |

| Time (d) | Sample position (Layers) | pH | ORP (mv) | DO (mg/L) | TDS (mg/L) | $SO_4^{2-}$ (mg/L) | $Cl^-$ (mg/L) | $HCO_3^-$ (mg/L) | $Na^++K^+$ (mg/L) | $Ca^{2+}$ (mg/L) | $Mg^{2+}$ (mg/L) |
|---|---|---|---|---|---|---|---|---|---|---|---|
| | Fine sandstone | 7.17 | 26.80 | 4.75 | 4663.35 | 2495.93 | 191.99 | 416.63 | 1119.79 | 289.16 | 35.66 |
| | Sandy mudstone | 7.20 | 52.28 | 4.69 | 4632.82 | 2494.04 | 192.28 | 454.17 | 1095.89 | 282.04 | 34.78 |
| | 2# coal | 7.17 | 89.60 | 4.71 | 4646.15 | 2479.76 | 191.38 | 466.14 | 1093.17 | 281.59 | 34.80 |
| | Medium sandstone | 7.17 | 118.50 | 5.59 | 4637.98 | 2495.87 | 191.73 | 448.23 | 1093.49 | 282.33 | 34.87 |
| | Mudstone | 7.16 | 109.25 | 6.27 | 4647.87 | 2502.82 | 192.18 | 435.51 | 1092.15 | 282.87 | 34.60 |
| | 3# coal | 7.15 | -62.52 | 3.43 | 4642.62 | 2396.11 | 196.54 | 470.74 | 1097.49 | 269.26 | 36.20 |
| | Caving rock | 7.17 | -31.25 | 3.26 | 4648.73 | 2394.40 | 195.15 | 447.26 | 1094.83 | 271.21 | 36.52 |
| | Fine sandstone | 7.17 | -36.95 | 2.97 | 4650.02 | 2486.73 | 201.42 | 476.40 | 1133.69 | 272.85 | 37.08 |
| 60 | Sandy mudstone | 7.15 | 35.90 | 4.30 | 4639.36 | 2397.92 | 195.42 | 500.29 | 1101.46 | 271.42 | 36.52 |
| | 2# coal | 7.15 | 71.50 | 4.58 | 4630.24 | 2535.13 | 205.17 | 484.52 | 1180.88 | 287.93 | 38.31 |
| | Medium sandstone | 7.16 | 68.45 | 4.56 | 4604.44 | 2385.55 | 195.55 | 426.00 | 1116.53 | 274.75 | 36.60 |
| | Mudstone | 7.16 | 102.90 | 4.40 | 4692.59 | 2383.29 | 195.13 | 468.20 | 1120.64 | 276.13 | 36.77 |
| | 3# coal | 7.14 | -79.28 | 3.53 | 4801.55 | 2500.22 | 196.78 | 503.77 | 1101.63 | 287.24 | 39.54 |
| | Caving rock | 7.14 | -79.45 | 3.49 | 4808.26 | 2537.63 | 197.88 | 522.70 | 1125.38 | 291.42 | 40.26 |
| | Fine sandstone | 7.14 | -81.45 | 3.57 | 4820.73 | 2541.65 | 197.45 | 520.84 | 1125.97 | 292.28 | 39.82 |
| 90 | Sandy mudstone | 7.13 | -65.20 | 3.76 | 4797.08 | 2553.55 | 200.04 | 486.45 | 1125.07 | 291.82 | 39.77 |
| | 2# coal | 7.15 | -28.65 | 3.34 | 4792.78 | 2463.65 | 193.47 | 463.08 | 1080.48 | 281.28 | 38.29 |
| | Medium sandstone | 7.14 | 44.90 | 3.79 | 4804.82 | 2542.25 | 198.21 | 511.95 | 1126.64 | 290.91 | 39.35 |
| | Mudstone | 7.13 | 69.90 | 4.40 | 4805.25 | 2551.60 | 214.48 | 474.51 | 1129.30 | 291.07 | 38.20 |
| 120 | 3# coal | 7.22 | -41.34 | 3.63 | 4846.79 | 2651.14 | 202.17 | 305.64 | 1098.53 | 292.76 | 43.06 |
| | Caving rock | 7.23 | -35.45 | 3.74 | 4842.23 | 2693.87 | 205.91 | 278.65 | 1104.91 | 292.74 | 43.20 |

| Time (d) | Sample position (Layers) | pH | ORP (mv) | DO (mg/L) | TDS (mg/L) | $SO_4^{2-}$ (mg/L) | $Cl^-$ (mg/L) | $HCO_3^-$ (mg/L) | $Na^++K^+$ (mg/L) | $Ca^{2+}$ (mg/L) | $Mg^{2+}$ (mg/L) |
|---|---|---|---|---|---|---|---|---|---|---|---|
| | Fine sandstone | 7.24 | -43.65 | 3.55 | 4858.57 | 2665.80 | 204.39 | 326.34 | 1106.96 | 293.72 | 43.45 |
| | Sandy mudstone | 7.25 | -6.26 | 3.47 | 4865.36 | 2632.13 | 202.80 | 337.34 | 1101.91 | 287.96 | 42.66 |
| | 2# coal | 7.26 | 32.45 | 3.62 | 4890.39 | 2623.75 | 198.86 | 421.49 | 1115.79 | 293.61 | 45.40 |
| | Medium sandstone | 7.30 | 41.45 | 3.56 | 4859.43 | 2621.00 | 200.53 | 397.27 | 1115.26 | 291.07 | 42.30 |
| | Mudstone | 7.31 | 68.50 | 4.36 | 4899.85 | 2612.23 | 199.99 | 339.09 | 1093.26 | 287.59 | 41.84 |
| 150 | 3# coal | 7.34 | -59.36 | 3.23 | 4938.81 | 2659.23 | 199.58 | 464.67 | 1135.31 | 307.30 | 44.35 |
| | Caving rock | 7.32 | -37.95 | 2.94 | 4955.32 | 2683.81 | 202.45 | 440.26 | 1142.13 | 308.35 | 43.00 |
| | Fine sandstone | 7.33 | -59.65 | 3.12 | 4928.66 | 2655.90 | 196.77 | 469.89 | 1135.57 | 308.55 | 43.22 |
| | Sandy mudstone | 7.23 | -33.28 | 2.50 | 4951.19 | 2654.90 | 198.24 | 388.85 | 1113.83 | 303.71 | 43.14 |
| | 2# coal | 7.38 | -18.75 | 2.46 | 4943.28 | 2646.24 | 198.25 | 428.17 | 1100.67 | 305.03 | 43.23 |
| | Medium sandstone | 7.32 | 1.75 | 2.47 | 4926.51 | 2634.85 | 196.81 | 464.49 | 1121.93 | 306.49 | 44.04 |
| | Mudstone | 7.53 | 57.60 | 3.57 | 4907.59 | 2642.87 | 198.01 | 472.70 | 1128.50 | 307.41 | 41.71 |
| 180 | 3# coal | 7.10 | -110.88 | 2.39 | 5013.97 | 2643.80 | 200.99 | 490.25 | 1129.85 | 304.28 | 44.46 |
| | Caving rock | 7.09 | -114.35 | 2.48 | 5003.48 | 2563.67 | 198.79 | 471.18 | 1110.89 | 305.39 | 43.44 |
| | Fine sandstone | 7.10 | -112.60 | 2.45 | 4994.02 | 2621.52 | 195.74 | 514.21 | 1128.42 | 313.12 | 45.11 |
| | Sandy mudstone | 7.09 | -101.18 | 2.38 | 4987.66 | 2599.96 | 200.32 | 466.17 | 1109.02 | 305.07 | 42.83 |
| | 2# coal | 7.09 | -109.25 | 2.60 | 5014.23 | 2661.37 | 200.67 | 469.74 | 1130.40 | 315.92 | 45.21 |
| | Medium sandstone | 7.11 | -107.00 | 2.57 | 4988.86 | 2638.69 | 202.19 | 536.35 | 1151.86 | 315.66 | 41.94 |
| | Mudstone | 7.18 | -49.95 | 2.51 | 4950.59 | 2623.99 | 199.16 | 512.10 | 1140.73 | 322.07 | 43.33 |
| 210 | 3# coal | 7.11 | -116.58 | 2.09 | 4992.82 | 2718.50 | 198.16 | 484.76 | 1125.26 | 312.44 | 43.01 |
| | Caving rock | 7.15 | -109.00 | 2.62 | 4978.54 | 2704.59 | 199.70 | 483.94 | 1120.08 | 312.76 | 43.57 |

| Time (d) | Sample position (Layers) | pH | ORP (mv) | DO (mg/L) | TDS (mg/L) | $SO_4^{2-}$ (mg/L) | $Cl^-$ (mg/L) | $HCO_3^-$ (mg/L) | $Na^++K^+$ (mg/L) | $Ca^{2+}$ (mg/L) | $Mg^{2+}$ (mg/L) |
|---|---|---|---|---|---|---|---|---|---|---|---|
| | Fine sandstone | 7.15 | -112.30 | 2.32 | 4955.75 | 2658.48 | 202.40 | 456.58 | 1114.27 | 311.73 | 42.80 |
| | Sandy mudstone | 7.12 | -115.56 | 2.58 | 4915.42 | 2667.67 | 195.68 | 477.09 | 1109.52 | 307.27 | 42.97 |
| | 2# coal | 7.08 | -117.50 | 2.59 | 4975.96 | 2635.58 | 195.40 | 441.09 | 1107.13 | 314.00 | 43.95 |
| | Medium sandstone | 7.05 | -111.00 | 3.33 | 4883.08 | 2645.32 | 196.74 | 460.19 | 1112.99 | 306.55 | 42.19 |
| | Mudstone | 7.05 | -67.30 | 2.54 | 4827.18 | 2677.36 | 195.61 | 455.96 | 1115.11 | 305.95 | 40.44 |
| | 3# coal | 7.13 | -137.58 | 1.56 | 4559.89 | 2490.64 | 195.68 | 461.05 | 1055.62 | 291.79 | 39.57 |
| | Caving rock | 7.14 | -141.80 | 1.59 | 4546.82 | 2532.87 | 197.18 | 459.73 | 1077.71 | 291.61 | 40.93 |
| | Fine sandstone | 7.11 | -128.80 | 1.38 | 4534.78 | 2566.34 | 196.96 | 412.37 | 1082.01 | 291.74 | 40.62 |
| 240 | Sandy mudstone | 7.19 | -113.54 | 1.95 | 4603.75 | 2571.26 | 198.29 | 389.39 | 1066.08 | 292.78 | 40.42 |
| | 2# coal | 7.20 | -79.50 | 2.23 | 4538.22 | 2533.45 | 203.52 | 376.90 | 1070.59 | 293.37 | 39.52 |
| | Medium sandstone | 7.22 | -101.40 | 2.80 | 4518.44 | 2521.75 | 200.60 | 462.61 | 1091.66 | 295.67 | 41.10 |
| | Mudstone | 7.26 | -62.40 | 2.47 | 4533.06 | 2515.92 | 197.61 | 378.92 | 1037.82 | 294.69 | 39.87 |
| | 3# coal | 7.31 | -138.54 | 1.38 | 4582.25 | 2509.86 | 197.57 | 370.93 | 1047.52 | 295.22 | 40.84 |
| | Caving rock | 7.27 | -144.05 | 1.53 | 4569.18 | 2512.47 | 197.02 | 398.32 | 1062.58 | 294.52 | 41.11 |
| | Fine sandstone | 7.27 | -131.85 | 1.94 | 4585.52 | 2478.51 | 194.86 | 405.17 | 1044.56 | 295.24 | 40.30 |
| 300 | Sandy mudstone | 7.31 | -111.06 | 1.88 | 4561.44 | 2509.92 | 195.97 | 400.49 | 1049.78 | 302.36 | 40.73 |
| | 2# coal | 7.28 | -93.25 | 2.37 | 4593.69 | 2511.99 | 197.41 | 357.61 | 1047.08 | 292.63 | 40.45 |
| | Medium sandstone | 7.30 | -99.80 | 2.01 | 4576.49 | 2490.56 | 197.52 | 399.94 | 1043.35 | 300.94 | 40.47 |
| | Mudstone | 7.34 | -64.95 | 2.33 | 4611.32 | 2514.49 | 205.02 | 406.69 | 1047.81 | 302.10 | 40.71 |
